# Report on Second International Conference on Natural Products for Cancer Prevention and Therapy Held in Kayseri, Turkey, 8–11 November 2017

**DOI:** 10.3390/nu10010008

**Published:** 2017-12-23

**Authors:** Mükerrem Betül Yerer, Anupam Bishayee

**Affiliations:** 1 Department of Pharmacology, Faculty of Pharmacy, University of Erciyes, Kayseri 38039, Turkey; 2 Department of Pharmaceutical Sciences, College of Pharmacy, Larkin University, Miami, FL 33169, USA

## 1. Preface

Scientific experts from eight countries gathered to share their views and experience on the latest research on natural products for cancer prevention and therapy. The traditionally used herbal medicines, medicinal plants, plant extracts, fractions, and phytochemicals for cancer prevention and therapy were discussed throughout the meeting. The scientific program comprised of 12 plenary lectures, 23 oral presentations, and 72 posters, providing an opportunity for more than 130 natural product scientists to present their research in three days. Abstracts for plenary talks, oral presentations, and posters were published as proceedings of the meeting in the special issue of Proceedings, Volume 1 and Issue 10 (http://www.mdpi.com/2504-3900/1/10). The aim of this biannual meeting was to foster discussion and disseminate the results of the research on natural products that are used for cancer prevention and therapy. During the meeting, the scientific committee members of the meeting who attended the conference had been selected as judges to evaluate all of the oral and poster presentations and the three best oral and poster presentation awards have been granted to the young scientists. The participants were able to network and engage in discussion for potential collaboration to advance our knowledge on utility of natural products for prevention and treatment of cancer. 

## 2. Summary of the Scientific Presentations

### 2.1. Plenary Lectures

The meeting was successfully focused on the natural products being investigated for their efficiency in several cancer types and for their potency in cancer prevention. Only the plenary lectures have been summarized here in this manuscript, and all of the other oral and poster presentations have been listed where the abstracts can be reached from http://www.mdpi.com/2504-3900/1/10. 

#### 2.1.1. Growth Factors Responsible from the Cancer Progress: Role of Natural Products

##### Mükerrem Betül Yerer 

Growth factors are one of the main factors responsible from the uncontrolled cell progress in cancer. Up to date many scientists have focused on these factors either as the marker or as the targets in several cancer types. Yerer has presented a plenary lecture on the natural products targeting these factors (Nerve growth factor (NGF), epidermal growth factor (EGF), hepatocyte growth factors (HGF), fibroblast growth factors (FGF), vascular endothelial growth factors (VEGF), platelet derived growth factor (PDGF), and transforming growth factor (TGF-β) (http://www.mdpi.com/2504-3900/1/10/979) [1].

#### 2.1.2. Natural Products for Cancer Prevention and Therapy: Progress, Pitfalls and Promise

##### Anupam Bishayee

The presentation of Bishayee highlighted studies on cancer preventive and therapeutic attributes of various naturally occurring agents and underlying mechanisms of action, with special emphasis on results reported from our laboratory. Current limitations, challenges, and future directions of research for successful cancer drug development based on natural products will also be discussed (http://www.mdpi.com/2504-3900/1/10/982) [2].

#### 2.1.3. Novel Anticancer Capacities of Saffron

##### Amr Amin 

Amr Amin has presented a plenary lecture on the anticancer effects of the saffron’s main active ingredient “safranal” against HCC using in vitro, in silico, and network analyses. In their studies, in addition to the unique and differential cell cycle arrest, safranal showed pro-apoptotic effect through activation of both intrinsic and extrinsic initiator caspases implicating ER stress-mediated apoptosis (http://www.mdpi.com/2504-3900/1/10/834) [3].

#### 2.1.4. Cardiac Glycosides as Novel Modulators of Cancer Cell Survival

##### Marc Diederich

This plenary lecture focused on Cardiac Glycosides (GCs) can be considered as pharmacological agents, allowing for cancer cells to switch from one cell death modality to another. All the findings encourage to further explore a potential for CGs in general as cancer cell death modulators alone or in combination with other targeted treatments (http://www.mdpi.com/2504-3900/1/10/972) [4].

#### 2.1.5. Ins and Outs of Flavonoids in Cancer Prevention vs. Cancer Therapy: A Lesson from Quercetin in Leukemia

##### Gian Luigi Russo, Maria Russo, Carmela Spagnuolo, Idolo Tedesco, Stefania Moccia

Russo et al. has critically reviewed the clinical and pre-clinical studies on the concept that polyphenols, being antioxidant compounds, can fight cancer. They suggest that a clear distinction must be done between the use of polyphenols, such as flavonoids, in cancer treatment versus cancer prevention, starting from adequate and specifically selected cellular models. As an example, he has present data on the potential application of quercetin against chronic lymphocytic leukemia (CLL) (http://www.mdpi.com/2504-3900/1/10/977) [5]. 

#### 2.1.6. Anticancer Potential of Flavones

##### Randolph RJ Arroo, Didem Şöhretoğlu, Demetrios A Spandidos, Vasilis P Androutsopoulos

Flavones are abundantly present in common fruits and vegetables, many of which have been associated with cancer prevention. Taking into account that no flavonoid based drugs are clinically used in cancer therapy, Randolph has focused on the flavones—which constitute a subgroup of the flavonoids—show some structural analogy with estrogen, and are known to interact with human estrogen receptors, either as agonist or as antagonist. Thus, whereas epidemiological and pre-clinical data seem to indicate a high potential for flavonoids, from the point of view of the pharmaceutical industry and drug developers, they are considered poor candidates (http://www.mdpi.com/2504-3900/1/10/975) [6]. 

#### 2.1.7. Resveratrol in Cancer Prevention and Treatment: Focusing on Molecular Targets and Mechanism of Action

##### Adriano Borriello

The relevance of these mechanisms and their translation in clinical therapy has been discussed in Borelli’s plenary lecture. Resveratrol and its mechanism of action has been emphasized by her in cancer cells and in experimental models of senescence, inflammation, obesity, and metabolic diseases. Its molecular targets act at different levels: (1) specific molecular pathways (like p53, NF-kappaB, PKC, PI3K, MDM2, LATS1, STK3 and several others); (2) epigenetic control of gene transcription through sirtuin activation; (3) cell division cycle and differentiation; (4) apoptosis and autophagy; and, (5) cellular redox homeostasis (http://www.mdpi.com/2504-3900/1/10/976) [7]. 

#### 2.1.8. Cynaropicrin: A Promising Natural Agent with Antitumor and Antiviral Activities

##### Mahmoud F. Elsebai, Jukka Hakkola, Mohamed Mehiri, Juana Diez

Human infection with HCV is currently recognized as the leading cause of hepatocellular carcinoma (HCC), which demands liver transplantation, which was estimated to result in ∼10,000 deaths in the US only in the year 2011. Elsebai has presented a plenary lecture on cynaropicrin as a potential agent for treatment and prevention of HCC by indirect way through inhibition of HCV and in a direct way evidenced by the many antitumor activities in literature (http://www.mdpi.com/2504-3900/1/10/974) [8]. 

#### 2.1.9. Relationship between Structure of Phenolics and Anticancer Activity

##### Müberra Koşar

Many phenolic compounds have been investigated for their potential use as cancer chemopreventive agents. Phenolic compounds consist of one or more hydroxyl substitution on the aromatic ring system. Koşar has emphasized that Cinnamic acid esters, such as caffeic acid phenethyl and benzyl esters, display selective antiproliferative activity against some types of cancer cells. Flavonoids consist of a large group of polyphenolic compounds having a benzo-𝛾-pyrone structure, and are ubiquitously present in plants. This structure can be responsible from the anticancer acitvities of these compounds (http://www.mdpi.com/2504-3900/1/10/978) [9]. 

#### 2.1.10. Pristimerin is a Promising Natural Product against Breast Cancer In Vitro and In Vivo through Apoptosis and the Blockage of Autophagic Flux

##### Buse Cevatemre, Konstantinos Dimas, Bruno Botta and Engin Ulukaya

Ulukaya has given a lecture on the pristimerin’s cytotoxic potential on particularly cancer stem cells (CSCs) should be much more important due to the CSCs’ recent role in recurrence of cancer. He has presented their studies on Pristimerin that has been shown to suppress the proliferation of various cancer cell lines at relatively lower concentrations, of which, the IC50 values are around 0.5–4 μM (http://www.mdpi.com/2504-3900/1/10/973) [10].

#### 2.1.11. Can Curcumin Be Employed to Promote the Integration of Oncology and Natural Products? 

##### Mutlu Demiray and Fatemeh Bahadori

Curcumin is multi-targeted molecule with pleotropic nature, which inhibits NF-κB and related proteins promoting effectiveness of tyrosine kinase inhibitors (TKIs). Demiray has presented their clinical studies with curcumin on adenoid cystic carcinoma where they have treated patients for 72 months by oral curcumin and eight months by i.v curcumin. Disease control rate was 89.3% (15/17), and no any grade III-IV toxicities was observed related to curcumin reflecting the clinical use of curcumin on adenoid cystic carcinoma patients (http://www.mdpi.com/2504-3900/1/10/980) [11]. 

#### 2.1.12. Therapeutic Potential of Black Pepper Compound for BRaf Resistant Melanoma

##### Neel M. Fofaria, Sharavan Ramachandran, and Sanjay K. Srivastava

Srivastava’s presentation mainly focused on the combination of BRAF inhibitors with Mcl-1 inhibitor such as piperlongumine may have therapeutic advantage to melanoma patients with acquired resistance to BRAF inhibitors alone or in combination with MEK1/2 inhibitors (http://www.mdpi.com/2504-3900/1/10/981) [12].

### 2.2. Oral and Poster Presentations

**Oral Presentations****Title****Authors****Link**Effect of Pomegranate Extract and Tangeretin on Specific Pathways in the Rat Breast Cancer Model Induced with DMBA [13].H. Fatih Gul et al. http://www.mdpi.com/2504-3900/1/10/983Synergistic Cytotoxic Effects of Resveratrol in Combination with Ceramide Metabolizing Enzymes in Ph + Acute Lymphoblastic Leukemia [14].Osman Oğuz et al. http://www.mdpi.com/2504-3900/1/10/984Characterization of cycloartane-type sapogenol derivatives for prostate cancer chemoprevention [15].Bilge Debelec-Butuner et al.http://www.mdpi.com/2504-3900/1/10/985Epibrassinolide treatment caused autophagy or apoptosis decision in a time-dependent manner through ER stress in colon cancer cells [16].Pınar Obakan-Yerlikaya et al. http://www.mdpi.com/2504-3900/1/10/986Determination of Silymarin molecule activity in colon cancer by AgNOR technique [17].Merve Alpay et al.http://www.mdpi.com/2504-3900/1/10/987The cytotoxic effect of *Lysimachia savranii* on the neuroblastoma cells [18].Gonca Dönmez et al. http://www.mdpi.com/2504-3900/1/10/988Autocrine Growth Hormone-triggered curcumin resistance abolished by NF-κB signaling pathway dependent on inflammatory cytokines and active polyamine catabolic machinery in MCF-7, MDA-MB-453 and MDA-MB-231 breast cancer cells [19].Ajda Çoker Gürkan et al.http://www.mdpi.com/2504-3900/1/10/989The effect of *Lysimachia savranii* on the migration of the breast cancer cells [20].Işıl Aydemir et al.http://www.mdpi.com/2504-3900/1/10/990Investigation of cytotoxic effect of *Origanum minutiflorum* on cancer cells [21].Oktay Özkan et al.http://www.mdpi.com/2504-3900/1/10/991Celastrol modulates lipid synthesis via PI3K/Akt/mTOR signaling axis to finalize cell death response in prostate cancer cells [22].Elif Damla Arisan et al. http://www.mdpi.com/2504-3900/1/10/992Investigation of the Effect of Paclitaxel and Pycnogenol on Mitochondrial Dynamics in Breast Cancer Therapy [23].Suna Sayğılı et al. http://www.mdpi.com/2504-3900/1/10/993Effects of curcumin on lipid peroxidation and antioxidant enzymes in kidney, liver, brain and testis of mice bearing Ehrlich Solid Tumor [24].Mustafa Nisari et al. http://www.mdpi.com/2504-3900/1/10/994Curcumin enhances the efficacy of 5-FU in Colo205 cell lines [25].Ebru Öztürk et al. http://www.mdpi.com/2504-3900/1/10/995Effect of a New Sapogenol Derivative (AG-07) on Cell Death via Necrosis [26].Yalcin Erzurumlu et al.http://www.mdpi.com/2504-3900/1/10/996Cytotoxic and Antiinflammatory Activity Guided Studies on *Plantago holosteum* Scop [27].Yasin Genc et al. http://www.mdpi.com/2504-3900/1/10/997Continuously monitoring the cytotoxicity of API-1, α-chaconine and α-solanine on human lung carcinoma A549 [28].Ebru Öztürk et al. http://www.mdpi.com/2504-3900/1/10/998The effects of α-chaconine on ER-α positive endometrium cancer cells [29].Ayşe Kübra Karaboğa Arslan et al. http://www.mdpi.com/2504-3900/1/10/999Investigation of apoptotic effect of sinapic acid in Hep3B and HepG2 human hepatocellular carcinoma cells [30].Canan Eroğlu et al.http://www.mdpi.com/2504-3900/1/10/1000Cytotoxic and Antioxidant Activity of four *Cousinia* Species of Stenocephalae Bunge. Section [31].Leyla Paşayeva et al. http://www.mdpi.com/2504-3900/1/10/1001Apoptotic effect of Ginnalin A on MDA-MB-231 and MCF7 human breast cancer cell lines [32].Ebru Avcı et al.http://www.mdpi.com/2504-3900/1/10/1002Cytotoxic effects of coumarin compounds imperatorin and osthole, alone and in combination with 5-fluorouracil in colon carcinoma cells [33].Ayşe Eken et al. http://www.mdpi.com/2504-3900/1/10/1003Screening of some Apiaceae and Asteraceae plants for their cytotoxic potential [34].Perihan Gürbüz et al.http://www.mdpi.com/2504-3900/1/10/1004Cyclodextrine Based Nanogels and Phase Solubility Studies of Flurbiprofen as a Chemopreventive Agent [35].Ayşe Nur Oktay et al. http://www.mdpi.com/2504-3900/1/10/1005**Poster Presentations**Effect of a synthesized compound against cancerous cell line and synthesis of copper ion incorporated 1-(3,4-diaminophenyl) ethanone-based hybrid nanoflowers [36].Burcu Somtürk Yılmaz et al. http://www.mdpi.com/2504-3900/1/10/1006Development of effective anticancer drug candidates against breast and colon cancers [37].Senem Akkoç et al.http://www.mdpi.com/2504-3900/1/10/1007Synthesis of copper ion incorporated aminoguanidine derivatives-based hybrid nanoflowers [38].Sevtap Çağlar Yavuz et al.http://www.mdpi.com/2504-3900/1/10/1008Evaluation of anti-proliferative and cytotoxic properties of chlorogenic acid against breast cancer cell lines by real time monitoring [39].Onur Bender et al. http://www.mdpi.com/2504-3900/1/10/1009Investigation of Apoptotic Effects of Usnic Acid on Hepatocellular Carcinoma [40].Beste Yurdacan et al.http://www.mdpi.com/2504-3900/1/10/1010*In vitro* Cytotoxic Effect Evaluation of *Dioscorea communis* (L.) Caddick & Wilkin Rhizome and Stem Extracts on Hepatocellular Carcinoma Cells [41].Ünal Egeli et al. http://www.mdpi.com/2504-3900/1/10/1011The Effect of Herbal Medicine on Neuroblastoma Cell Line in Culture [42].Büşra Şen et al.http://www.mdpi.com/2504-3900/1/10/1012The foods containing miR-193b may inhibit the growth of breast cancer cells [43].Dilek Asci Celik et al.http://www.mdpi.com/2504-3900/1/10/1013Is the dietary miR-193b a novel cell cycle arresting source for breast carcinoma? [44].Nilgun Gurbuz et al.http://www.mdpi.com/2504-3900/1/10/1014The effects of Wortmannin and EGCG and combined treatments on MDA-MB-231 breast cancer cell lines via inactivation of PI3K signaling pathway [45].Elgin Turkoz Uluer et al. http://www.mdpi.com/2504-3900/1/10/1015The effects of Paclitaxel and Metformin and combined treatments on TLR signaling pathway on MDA-MB-231 breast cancer cell lines [46].Melike Ozgul et al.http://www.mdpi.com/2504-3900/1/10/1016Inhibition of telomerase activity by cucurbitacin I in colon cancer cell line, LS174T [47].Emir Tosun et al. http://www.mdpi.com/2504-3900/1/10/1017Effect of cucurbitacin I on proliferation and migration in colorectal cancer cell line, LS174T [48].Emir Tosun et al. http://www.mdpi.com/2504-3900/1/10/1018*In vitro* anticancer and cytotoxic activities of some plant extracts on HeLa and Vero cell lines [49].Fulya Tugba Artun et al. http://www.mdpi.com/2504-3900/1/10/1019Anticancer Effects of Oleocanthal and *Pinus Pinaster* on Breast Cancer Cell in Culture [50].Mahmud Özkut et al.http://www.mdpi.com/2504-3900/1/10/1020Antiproliferative and Apoptotic Effects of the Medicinal Plants on Breast Cancer Cell Lines [51].Pınar Kılıçaslan Sönmez et al.http://www.mdpi.com/2504-3900/1/10/1021The role of trophoblastic stem cells conditioned media on JAR cell culture [52].Hilal Kabadayı et al.http://www.mdpi.com/2504-3900/1/10/1022The effect of pycnogenol and paclitaxel on DNA damage in human breast cancer cell line [53].Hülya Birinci et al.http://www.mdpi.com/2504-3900/1/10/1023Investigation of the effects of paclitaxel and pycnogenol on inflammatory response (PTX3, BDNF, IGF2R) in human breast cancer cell line [54].Hülya Birinci et al.http://www.mdpi.com/2504-3900/1/10/1024Is There Any Protective Effect of Pomegranate and Tangeretin on the DMBA-Induced Rat Breast Cancer Model? [55].H. Fatih Gul et al. http://www.mdpi.com/2504-3900/1/10/1025The neurotoxic effects of *Origanum minutiflorum* [56].İsmail Sari et al.http://www.mdpi.com/2504-3900/1/10/1026The Cytotoxic and Apoptotic Effects of Usnic Acid on Prostate Cancer versus Normal Cells [57].Işıl Ezgi Eryılmaz et al.http://www.mdpi.com/2504-3900/1/10/1027Antiproliferative Effect of Methanolic Extract of *Linum arboretum* on A549 Cells [58].Ozgur Vatan et al.http://www.mdpi.com/2504-3900/1/10/1028Investigation of *in vitro* Cytotoxic Effects of *Montivipera xanthina* on Healthy and Cancer Human Lung Cell Lines [59].Huzeyfe Huriyet et al. http://www.mdpi.com/2504-3900/1/10/1029Development and Characterization of Paclitaxel-loaded PLGA Nanoparticles and Evaluation of Cytotoxicity on MCF-7 cell line by MTT Assay [60].Merve Çelik Tekeli et al.http://www.mdpi.com/2504-3900/1/10/1030Effects of Fulvic Acid on Different Cancer Cell Lines [61].S. Kerem Aydin et al.http://www.mdpi.com/2504-3900/1/10/1031Antioxidant, antibacterial and antiproliferative activities of Turkish rhubarb (*Rheum palmatum* L.) leaf extracts [62].Mehmet Berköz et al.http://www.mdpi.com/2504-3900/1/10/1032The Effect of Herbal Medicine on Colon Cancer Cells in Culture [63].Pelin Toros et al.http://www.mdpi.com/2504-3900/1/10/1033The Effect of Herbal Medicine on Prostate Cancer Cells in Culture [64].Pelin Toro et al.http://www.mdpi.com/2504-3900/1/10/1034Determination of Antioxidant Capacity, Phenolic Acid Composition and Antiproliferative Effect Associated with Phenylalanine Ammonia Lyase (PAL) Activity in Some Plants Naturally Growing under Salt Stress [65].Seda Şirin et al. http://www.mdpi.com/2504-3900/1/10/1035Development and Characterization of Paclitaxel-loaded PLGA Nanoparticles and Cytotoxicity Assessment by MTT assay on A549 cell line [66].Sedat Ünal et al.http://www.mdpi.com/2504-3900/1/10/1036Evaluation of in vitro anti-proliferative activity of St. John’s wort (*Hypericum perforatum* Linn.) plant extract on cervix adenocarcinoma [67].Rana Kavurmacı et al.http://www.mdpi.com/2504-3900/1/10/1037The cytotoxic effect of *Annona muricata* leaf extract on triple negative breast cancer cell line [68].Rana Kavurmacı et al.http://www.mdpi.com/2504-3900/1/10/1038Cytotoxic activity of *Achillea coarctata* Poir. Extract [69].Sevil Albayrak et al.http://www.mdpi.com/2504-3900/1/10/1039Cytotoxic activity of Endemic *Astragalus argaeus* Boiss. from Turkey [70].Sevil Albayrak et al.http://www.mdpi.com/2504-3900/1/10/1040Lactic Acid Bacteria Mediated Apoptosis Induction: Natural way of colon cancer cells’ inhibition [71].Şebnem Kurhan et al.http://www.mdpi.com/2504-3900/1/10/1041Synthesized a new organic compound’s cytotoxic activity quantum mechanics calculations and docking studies [72].Senem Akkoç et al. http://www.mdpi.com/2504-3900/1/10/1042Anticancer Activity of *Centaurea babylonica* L. [73].Elif Dündar et al.http://www.mdpi.com/2504-3900/1/10/1043Cytotoxic Effects of Functional Foods *Momordica charantia* L. and *Lycium barbarum* L. Extracts on Prostate Cancer Cells [74].Guzide Satir Basaran et al. http://www.mdpi.com/2504-3900/1/10/1044Cytotoxic Effects of Kynurenic acid and Quinaldic acid in Hepatocellular Carcinoma (HepG2) cell line [75].Pınar Atalay Dündar et al.http://www.mdpi.com/2504-3900/1/10/1045The Effects of Benzoxasol Derivate Compounds in Breast Cancer Cells [76].Funda Kosova et al.http://www.mdpi.com/2504-3900/1/10/1046Potential Cytotoxic Activity of *Psephellus pyrrhoblepharus* Extracts [77].Pelin Taştan et al.http://www.mdpi.com/2504-3900/1/10/1047Screening of *Onosma* species for Cytotoxic Activity [78].Özge Güzel et al.http://www.mdpi.com/2504-3900/1/10/1048Apoptotic Effects of *Mount Bulgar Viper (Montivipera bulgardaghica)* PLA2 and SVMPs Venom Peptide fractions on HeLa and A549 Cancer Cells [79].Yalcin Erzurumlu et al.http://www.mdpi.com/2504-3900/1/10/1049Turkish Propolis Extract Increases Apoptosis via Induction of Mitochondrial Membrane Potential Loss in MCF-7 Cells [80].Sema Misir et al. http://www.mdpi.com/2504-3900/1/10/1050The Effect of Gilaburu (*Viburnum opulus*) Juice on Ehrlich Ascites Tumor (EAT) Cell Culture [81].Özge Al et al.http://www.mdpi.com/2504-3900/1/10/1051Synthesis and characterizations of folate-conjugated PLGA-PEG nanoparticles loaded with dual agents [82].Yüksel Öğünç et al. http://www.mdpi.com/2504-3900/1/10/1052Selective cytotoxic activity of *Scutellaria* species [83].Zeynep Dogan et al.http://www.mdpi.com/2504-3900/1/10/1053The Antiproliferative Effect of Alpha Tocopherol in F98 Cell Culture [84].Remzi Soner Cengiz et al.http://www.mdpi.com/2504-3900/1/10/1054Analysis of the Cytotoxic Effects of *Eryngium billardieri* Delar. Extracts on MCF7 Cell Line [85].Leyla Paşayeva et al.http://www.mdpi.com/2504-3900/1/10/1055Cytotoxic Effects of *Alchemilla mollis* (Buser) Rothm. Extracts on MCF 7 cell line [86].Selen İlgün et al.http://www.mdpi.com/2504-3900/1/10/1056Comparative Evaluation of the cytotoxic effects of stem and flower extracts of *Rhaponticoides iconiensis* (Hub.-Mor.) M.V.Agab. & Greuter [87].Eren Demirpolat et al. http://www.mdpi.com/2504-3900/1/10/1057Goji berry fruit extract suppresses cell proliferation of breast cancer cells by inhibiting EGFR/ERK signaling [88].Hatice Bekci et al. http://www.mdpi.com/2504-3900/1/10/1058Biologically transformed Propolis Exhibits Cytotoxic Effect on A375 Malignant Melanoma Cells in vitro [89].Hikmet Memmedov et al.http://www.mdpi.com/2504-3900/1/10/1059*Rheum ribes* extract increase the expression level of miR-200 family in human colorectal cancer cells [90].Ilknur Cinar et al. http://www.mdpi.com/2504-3900/1/10/1060Potential effects of *Liquidambar orientalis* Mill. against HT-29 and HCT-116 cell lines [91].Sumeyra Cetinkaya et al.http://www.mdpi.com/2504-3900/1/10/1061The Effect of Tocopherol-α On the Cell Viability in Caco-2 Cell Line [92].Ayşenur Gök et al.http://www.mdpi.com/2504-3900/1/10/1062In vitro Antioxidant and Anticancer Activities of Some Local Plants from Bolu Province of Turkey [93].Kadriye Nur Kasapoğlu et al.http://www.mdpi.com/2504-3900/1/10/1063Survey of the apoptotic effect of Ginnalin A on Hep3B human hepatocellular carcinoma cell line [94]. Pınar Özden et al.http://www.mdpi.com/2504-3900/1/10/1064Ameliorative effects of Carvacrol on Cyclophosphamide-induced testis damage and oxidative stress [95].Mustafa Cengiz et al. http://www.mdpi.com/2504-3900/1/10/1066Synthesis of Anthocyanin-rich Red Cabbage Nanoflowers and Their Antimicrobial and Cytotoxic Properties [96].Suheyl Furkan Konca et al.http://www.mdpi.com/2504-3900/1/10/1067Cytotoxic potentials of some Asteraceae plants from Turkey on HeLa cell line [97].Kübra Uzun et al.http://www.mdpi.com/2504-3900/1/10/1068The Role of Lidocaine in the Dunning Model Rat Prostate Cancer Cells: Cell Kinetics and Motility [98].Esma Purut et al. http://www.mdpi.com/2504-3900/1/10/1069*Pelargonium endlicherianum* Fenzl. Root extract suppresses cell proliferation of prostate cancer cells [99].Selda Eren et al.http://www.mdpi.com/2504-3900/1/10/1070Assessment of antioxidant and cytotoxic activity of known antioxidants compared to neopterin [100].Gözde Girgin et al.http://www.mdpi.com/2504-3900/1/10/1071Comparison of radical scavenging and cytotoxic activities of well-known non-enzymatic antioxidants [101].Suna Sabuncuoğlu et al.http://www.mdpi.com/2504-3900/1/10/1072A Study on the Synthesis and Anticancer Activities of Novel 6-Methoxy Flavonyl Piperazine Derivatives [102].Meltem Ceylan-Ünlüsoy et al. http://www.mdpi.com/2504-3900/1/10/1073Effect of Paclitaxel Loaded Chitosan Nanoparticles and Quantum Dots on Breast Cancer [103].Gülen Melike Demir et al.http://www.mdpi.com/2504-3900/1/10/1074Turkish Medicinal Plants Used in Cancer Treatment and Evaluation of Plant Usage in the Oncology Clinic of the İstanbul University Faculty of Medicine [104].Büşra Teke et al. http://www.mdpi.com/2504-3900/1/10/1075Is Acteosid Effects on Colon Cancer Stem Cells Via Inflamation and/or Apoptosis? [105].Fatma Firat et al.http://www.mdpi.com/2504-3900/1/10/1076Analysis of the Cytotoxic Effects of *Achillea millefolium* L. Extracts on MCF7 Cell Line [106].Esra Köngül et al.http://www.mdpi.com/2504-3900/1/10/1077

## 3. Author Affiliations

- Abdurrahim Kocyigit, Department of Medical Biochemistry, Faculty of Medicine, Bezmialem Vakif University, Sarıyer, Turkey
- Ademi Fahri Pirhan, Department of Biology, Faculty of Science, Ege University, Izmir, Turkey
- Adnan Ayhanci, Biology Department, Art and Science Faculty, Eskişehir Osmangazi University, Eskişehir, Turkey
- Adriana Borriello, Department of Biochemistry, Biophysics and General Pathology, University of Campania “L. Vanvitelli”, Naples, Italy
- Ahmet Baysar, Department of Chemical Engineering, Inonu University, Malatya, Turkey
- Ahmet Cumaoglu, Department of Biochemistry, Faculty of Pharmacy, Erciyes University, Kayseri, Turkey
- Ahmet Savran, Department of Arts and Sciences, Faculty of Medicine, Niğde Ömer Halisdemir University, Niğde, Turkey
- Ajda Çoker-Gürkan, Department of Molecular Biology and Genetics, Istanbul Kultur University, Atakoy Campus, Istanbul, Turkey
- Ali Karagoz, Department of Molecular Biology and Genetics, Faculty of Science, Istanbul University, Istanbul, Turkey
- Amr Amin, Biology Department, UAE University, Abu Dhabi, United Arab Emirates
- Anupam Bishayee, Department of Pharmaceutical Sciences, College of Pharmacy, Larkin University, Miami, FL, USA
- Arzu Atalay, Biotechnology Institute, Ankara University, Ankara, Turkey
- Asuman Bozkir, Department of Pharmaceutical Technology, Faculty of Pharmacy, Ankara University, Ankara, Turkey
- Ayhan Altıntaş, Department of Pharmacognosy, Faculty of Pharmacy, Anadolu University, Eskişehir, Turkey
- Aynur Işık, Department of Molecular Biology and Genetics, Faculty of Science, Gazi University, Ankara, Turkey
- Ayse Baldemir, Department of Pharmaceutical botany, Faculty of Pharmacy, Erciyes University, Kayseri, Turkey
- Ayse Nalbantsoy, Department of Bioengineering, Faculty of Engineering, Ege University, Izmir, Turkey
- Aysun Adan, Molecular Biology and Genetics, Faculty of Life and Natural Sciences, Abdullah Gul University, Kayseri, Turkey
- Aysun Ökçesiz, Department of Pharmaceutical Toxicology, Faculty of Pharmacy, Erciyes University, Kayseri, Turkey
- Ayşe Eken, Department of Pharmaceutical Toxicology, Faculty of Pharmacy, Erciyes University, Kayseri, Turkey
- Ayşe Kübra Karaboğa Arslan, Department of Pharmacology, Faculty of Pharmacy, Erciyes University, Kayseri, Turkey
- Ayşe Nur Oktay, Department of Pharmaceutical Technology, Faculty of Pharmacy, Gazi University, Ankara, Turkey
- Ayşe Zeynep Ünal, Toxicology Department, Faculty of Pharmacy, Hacettepe University, Ankara, Turkey
- Ayşenur Gök, Ankara Unıversıty, Faculty of Veterinary Medicine, Ankara, Turkey
- Basseem Radwan, Department of Pharmacology, Faculty of Pharmacy, Erciyes University, Kayseri, Turkey
- Bayram Goçmen, Zoology Section, Department of Biology, Faculty of Science, Ege University, Izmir, Turkey
- Belma Aslım, Gazi University, Faculty of Science, Department of Biology, Teknikokullar, Ankara, Turkey
- Benjamin-Florian Hempel, Institut für Chemie, Technische Universitat Berlin, Strasse des 17. Juni 124, Berlin, Germany
- Beraat Özçelik, Department of Food Engineering, Faculty of Chemical and Metallurgical Enginerring, Istanbul Technical University, Turkey
- Berrin Tunca, Medical Biology Department, Faculty of Medicine, Uludag University, Bursa, Turkey
- Beste Yurdacan, Medical Biology Department, Faculty of Medicine, Uludag University, Gorukle, Bursa, Turkey
- Bijen Kıvçak, Deparment of Pharmacognosy, Faculty of Pharmacy, Ege University, İzmir, Turkey
- Bilge Debelec-Butuner, Department of Pharmaceutical Biotechnology, Faculty of Pharmacy, Ege University, Izmir, Turkey
- Bruno Botta, Dipartimento di Chimica e Tecnologie del Farmaco, Sapienza University of Roma, piazzale Aldo Moro 5, Roma, Italy
- Burak Durmaz, Department of Medical Biochemistry, Ege University, Turkey
- Burcu Somtürk Yılmaz, Department of Chemistry, Faculty of Sciences, Erciyes University, Kayseri, Turkey
- Burçin Türkmenoğlu, Department of Chemistry, Faculty of Sciences, Erciyes University, Kayseri, Turkey
- Buse Cevatemre, Department of Biology, Faculty of Arts and Sciences, Uludag University, Bursa, Turkey
- Büşra Şen, Department of Histology and Embryology, Faculty of Medicine, Celal Bayar University, Manisa, Turkey
- Büşra Teke, Faculty of Pharmacy, İstanbul University, Istanbul, Turkey
- Canan Eroğlu, Department of Medical Biology, Meram Faculty of Medicine, Necmettin Erbakan University, Konya, Turkey
- Canan Türkoğlu, Department of Biology, Faculty of Art and Life Sciences, Manisa Celal Bayar University, Manisa, Turkey
- Carmela Spagnuolo, Institute of Food Sciences, National Research Council, Avellino, Italy
- Çiğdem Yücel, Erciyes University Faculty of Pharmacy Department of Pharmaceutical Technology
- Damla Akogullari, Faculty of Medicine, Department of Histology & Embryology, Manisa Celal Bayar University, Manisa, Turkey
- Daniel Petras, Institut für Chemie, Technische Universitat Berlin, Strasse des 17. Juni 124, Berlin, Germany
- Demetrios A. Spandidos, Department of Toxicology, Medical School, University of Crete, Crete GR, Greece
- Didar Tasdemir, Department of Analytical Chemistry, Faculty of Pharmacy, Erciyes University, Kayseri, Turkey
- Didem Şöhretoğlu, Faculty of Pharmacy, Hacettepe Unıversıty, Ankara, Turkey
- Dilek Asci Celik, Department of Medical Biology, School of Medicine, Suleyman Demirel University, Isparta, Turkey
- Dilek Ceylan, Genome and Stem Cell Center, University of Erciyes, Kayseri, Turkey
- Ebru Avcı, Department of Medical Biology, Meram Faculty of Medicine, Necmettin Erbakan University, Konya, Turkey
- Ebru Öztürk, Department of Pharmacology, Faculty of Pharmacy, Erciyes University, Kayseri, Turkey
- Efe Kurtdede, Ankara, Turkey
- Elgin Turkoz Uluer, Department of Histology & Embryology, Faculty of Medicine, Manisa Celal Bayar University, Manisa, Turkey
- Elif Damla Arısan, Department of Molecular Biology and Genetics, Istanbul Kultur University, Atakoy Campus, Istanbul, Turkey
- Elif Dündar, Department of Pharmaceutical Botany, Graduate School of Health Sciences, Anadolu University, Eskişehir, Turkey
- Emin Sarıpınar, Department of Chemistry, Faculty of Sciences, Erciyes University, Kayseri, Turkey
- Emine Akalın Uruşak, Faculty of Pharmacy, İstanbul University, Istanbul, Turkey
- Emir Tosun, Department of Chemical Engineering, Inonu University, Malatya, Turkey
- Engin Ulukaya, Department of Clinical Biochemistry, Faculty of Medicine, Istinye University, Istanbul, Turkey
- Ercan Kurar, Department of Medical Biology, Meram Faculty of Medicine, Necmettin Erbakan University, Konya, Turkey
- Ercüment Ölmez, Faculty of Medicine, Department of Pharmacology, Celal Bayar University, Manisa, Turkey
- Erdal Bedir, Department of Bioengineering, Faculty of Engineering, Izmir Institute of Technology, Izmir, Turkey
- Erem Bilensoy, Hacettepe University Faculty of Pharmacy Department of Pharmaceutical Technology
- Eren Demirpolat, Department of Pharmacology, Faculty of Pharmacy, Erciyes University, Kayseri, Turkey
- Erkan Yilmaz, Biotechnology Institute, Ankara University, Ankara, Turkey
- Eser Yıldırım Sözmen, Department of Medical Biochemistry, Ege University, Turkey
- Esma Purut, Department of Biology, Faculty of Science, University of Istanbul, Istanbul, Turkey
- Esra Köngül, Department of Pharmacognosy, Faculty of Pharmacy, Erciyes University, Kayseri, Turkey
- Esra Küpeli Akkol, Department of Pharmaceutical Technology, Faculty of Pharmacy, Gazi University, Ankara, Turkey
- Evren Demircan, Department of Food Engineering, Faculty of Chemical and Metallurgical Enginerring, Istanbul Technical University, Turkey
- Ezgi Balkan, Department of Medical Biochemistry, Faculty of Medicine, Bezmialem Vakif University, Turkey
- Fatemeh Bahadori, Department of Pharmaceutical Biotechnology, Faculty of Pharmacy, Bezmialem Vakif University, Istanbul, Turkey
- Fatih Çöllü, Biology, Faculty of Science and Literature, Manisa Celal Bayar University, Manisa, Turkey
- Fatma Esin Kırık, Department of Medicinal Microbiology, Faculty of Medicine, Niğde Ömer Halisdemir University, Niğde, Turkey
- Fatma Firat, Department of Histology and Embryology, Faculty of Medicine, Manisa Celal Bayar University, Manisa, Turkey
- Feyzan Özdal Kurt, Department of Biology, Faculty of Art and Life Sciences, Manisa Celal Bayar University, Manisa, Turkey
- Fulya Tugba Artun, Institute of Science, Istanbul University, Istanbul, Turkey
- Funda Karbancıoğlu-Güler, Department of Food Engineering, Faculty of Chemical and Metallurgical Enginerring, Istanbul Technical University, Sarıyer, Turkey
- Funda Kosova, Faculty of Health Science, Celal Bayar University, Manisa, Turkey
- Funda Nuray Yalçın, Pharmacognosy Dept., Faculty of Pharmacy, Hacettepe University, Ankara, Turkey
- Gamze Güney Eskiler, Medical Biology Department, Faculty of Medicine, Sakarya University, Sakarya, Turkey
- Gian Luigi Russo Russo, Institute of Food Sciences, National Research Council, Avellino, Italy
- Gonca Dönmez, Department of Medicinal Biology, Faculty of Medicine, Niğde Ömer Halisdemir University, Niğde, Turkey
- Gorkem Kısmalı, Ankara, Turkey
- Gökçe Şeker Karatoprak, Erciyes University Faculty of Pharmacy Department of Pharmacognosy
- Gözde Girgin, Toxicology Department, Faculty of Pharmacy, Hacettepe University, Ankara, Turkey
- Gul Ozcan, Department of Biology, Faculty of Science, Istanbul University, Istanbul, Turkey
- Gulay Melikoglu, Department of Pharmacognosy, Faculty of Pharmacy, Istanbul University, Istanbul, Turkey
- Guzide Satır Basaran, Department of Biochemistry, Faculty of Pharmacy, Erciyes University, Kayseri, Turkey
- Gülen Melike Demir, Department of Pharmaceutical Technology, Faculty of Pharmacy, Gazi University, Ankara, Turkey
- Güliz Armagan, Department of Biochemistry, Faculty of Pharmacy, Ege University, İzmir, Turkey
- Gülşah Albayrak, Department of Histology and Embryology, Faculty of Medicine, Celal Bayar University, Manisa, Turkey
- Gülşah Çeçener, Medical Biology Department, Faculty of Medicine, Uludag University, Gorukle, Bursa, Turkey
- Gülşen Akalın Çiftçi, Department of Biochemistry, Faculty of Pharmacy, Anadolu University, Eskişehir, Turkey
- H. Fatih Gul, Department of Medical Biochemistry, Faculty of Medicine, Firat University, Elazig, Turkey
- H. Gul Dursun, Medical Biology Department, Meram Medical Faculty, Necmettin Erbakan University, Konya, Turkey
- H. Seda Vatansever, Department of Histology and Embryology, Faculty of Medicine, Manisa Celal Bayar University, Manisa, Turkey
- Hakkı Taştan, Department of Biology, Faculty of Science, Gazi University, Ankara, Turkey
- Harun Ülger, School of Medicine, Department of Anatomy, Erciyes University, Kayseri, Turkey
- Hasibe Vural, Department of Medical Biology, Meram Faculty of Medicine, Necmettin Erbakan University, Konya, Turkey
- Hatice Bekci, Department of Food Engineering, Engineering Faculty, Erciyes University, Kayseri, Turkey
- Hatice Kalkan Yıldırım, Faculty of Engineering Department of Food Engineering, Ege University, Turkey
- Hatice Susar, Department of Anatomy, Faculty of Medicine, Erciyes University, Kayseri, Turkey
- Hatice Yildirim, Department of Molecular Biology and Genetic, Balikesir University, Balikesir, Turkey
- Hikmet Memmedov, Department of Medical Biochemistry, Ege University, Turkey
- Hilal Kabadayı, Department of Histology and Embryology, School of Medicine, Manisa Celal Bayar University, Manisa, Turkey
- Hulusi Malyer, Botany Department, Faculty of Science and Art, Uludag University, Bursa, Turkey
- Huzeyfe Huriyet, Medical Biology Department, Faculty of Medicine, Uludag University, Bursa, Turkey
- Hülya Birinci, Department of Histology and Embryology, Faculty of Medicine, Celal Bayar University, Manisa, Turkey
- Hanifi Ozercan, Department of Medical Pathology, Faculty of Medicine, Firat University, Elazıg, Turkey
- Ibrahim Turan, Department of Genetic and Bioengineering, Faculty of Engineering and Natural Sciences, GumushaneUniversity, Gumushane, Turkey
- Iclal Saracoglu, Department of Pharmacognosy, Faculty of Pharmacy, Hacettepe University, Turkey
- Ilknur Cinar, Medical Biology Department, Meram Medical Faculty, Necmettin Erbakan University, Konya, Turkey
- Ismail Hakki Akgun, Department of Bioengineering, Faculty of Engineering, Ege University, Izmir, Turkey
- Ismail Ocsoy, Department of Analytical Chemistry, Faculty of Pharmacy, Erciyes University, Kayseri, Turkey
- Işıl Aydemir, Faculty of Medicine, Niğde Ömer Halisdemir University, Niğde, Turkey
- Işıl Ezgi Eryılmaz, Medical Biology Department, Faculty of Medicine, Uludag University, Bursa, Turkey
- İbrahim Çakir, Department of Food Engineering, Faculty of Engineering and Architecture, Abant İzzet Baysal University, Bolu, Turkey
- İbrahim Tuğlu, Department of Histology and Embryology, Medical of Faculty, Celal Bayar University, Manisa, Turkey
- İlhan Özer İlhan, Department of Chemistry, Faculty of Sciences, Erciyes University, Kayseri, Turkey
- İsmail Sari, Department of Medicinal Biochemistry, Faculty of Medicine, Niğde Ömer Halisdemir University, Niğde, Turkey
- İsmail Tuncer Değim, Department of Pharmaceutical Technology, Faculty of Pharmacy, Biruni University, Topkapı, İstanbul, Turkey
- Juana Diez, Department of Experimental and Health Sciences, Universitat Pompeu Fabra, Barcelona, Spain
- Jukka Hakkola, Pharmacology and Toxicology Unit, Institute of Biomedicne, University of Oulu, Oulu, Finland
- Kaan Adacan, Department of Molecular Biology and Genetics, Istanbul Kultur University, Atakoy Campus, Istanbul, Turkey
- Kadriye Nur Kasapoğlu, Department of Food Engineering, Faculty of Chemical and Metallurgical Enginerring, Istanbul Technical University, Turkey
- Kamil Vural, Department of Medicinal Pharmacology, Faculty of Medicine, Manisa Celal Bayar University, Manisa, Turkey
- Kemal Özbilgin, Department of Histology and Embryology, Faculty of Medicine, Celal Bayar University, Manisa, Turkey
- Kemal Sami Korkmaz, Department of Bioengineering, Faculty of Engineering, Ege University, Izmir, Turkey
- Konstantinos Dimas, Department of Pharmacology, Faculty of Medicine, University of Thessaly, Larissa, Greece
- Kübra Uzun, Pharmacognosy Dept., Faculty of Pharmacy, Erciyes University, Kayseri, Turkey
- Latife Merve Oktay, Faculty of Medicine Department of Medical Biology, Ege University, Turkey
- Leyla Paşayeva, Department of Pharmacognosy, Faculty of Pharmacy, Erciyes University, Kayseri, Turkey
- Mahmoud F. Elsebai, Pharmacognosy Department, Faculty of Pharmacy, Mansoura University, Mansoura, Egypt
- Mahmud Özkut, Department of Histology & Embryology, Faculty of Medicine, Manisa Celal Bayar University, Manisa, Turkey
- Marc Diederich, College of Pharmacy, Seoul National University, Seoul, Korea
- Maria usso, Institute of Food Sciences, National Research Council, Avellino, Italy
- Mehmet Berköz, Department of Pharmaceutical Biotechnology, Faculty of Pharmacy, Yuzuncu Yıl University, Van, Turkey
- Mehmet İbrahim Tuğlu, Department of Histology and Embryology, Faculty of Medicine, Manisa Celal Bayar University, Manisa, Turkey
- Mehmet Zülfü Yildiz, Zoology Section, Department of Biology, Faculty of Arts and Science, Adıyaman University, Adıyaman, Turkey
- Mehtap Nisari, Department of Anatomy, Faculty of Medicine, Erciyes University, Kayseri, Turkey
- Melike Ozgul, Department of Histology & Embryology, Faculty of Medicine, Manisa Celal Bayar University, Manisa, Turkey
- Meltem Ceylan-Ünlüsoy, Department of Pharmaceutical Chemistry, Faculty of Pharmacy, Ankara University, Ankara, Turkey
- Mert Burak Ozturk, Department of Bioengineering, Faculty of Engineering, Ege University, Izmir, Turkey
- Mert Ilhan, Department of Pharmaceutical Technology, Faculty of Pharmacy, Biruni University, İstanbul, Turkey
- Merve Alpay, Department of Biochemistry, Faculty of Medicine, Duzce University, Düzce, Turkey
- Merve Çelik Tekeli, Erciyes University Faculty of Pharmacy Department of Pharmaceutical Technology
- Merve Çelik, Department of Molecular Biology and Genetics, Faculty of Science and Letters, Istanbul Kultur University, Istanbul, Turkey
- Merve Karaman, Department of Biology, Balikesir University, Balikesir, Turkey
- Merve Uğur, Department of Molecular Biology and Genetics, Faculty of Science and Letters, Istanbul Kultur University, Istanbul, Turkey
- Metin Yıldırım, Department of Biochemistry, Faculty of Pharmacy, Mersin University, Mersin, Turkey
- Mirosław Krośniak, Department of Food Chemistry and Nutrition, Medical College, Jagiellonian University, Krakow, Poland
- Mohamed Mehiri, Nice, France
- Mustafa Cengiz, Department of Mathematics and Science Education, Education Faculty, Siirt University, Siirt, Turkey
- Mustafa Nisari, Department of Nutrition and Dietetics, Faculty of Health Sciences, University of Nuh Naci Yazgan, Kayseri, Turkey
- Mustafa Öztatlıcı, Department of Histology and Embryology, Faculty of Medicine, Manisa Celal Bayar University, Manisa, Turkey
- Mutlu Demiray, Department of Medical Oncology, KTO Karatay University, Konya, Turkey
- Müberra Koşar, Faculty of Pharmacy, Department of Pharmacognosy, Eastern Mediterranean University, Gazimağusa, North Cyprus via Mersin 10, Turkey
- Mükerrem Betül Yerer, Department of Pharmacology, Faculty of Pharmacy, Erciyes University, Kayseri, Turkey
- Müzeyyen Demirel, Department of Pharmaceutical Technology, Faculty of Pharmacy, Anadolu University, Tepebaşı, Turkey
- N. Nalan İmamoğlu, Department of Basic Sciences, Faculty of Pharmacy, Erciyes University, Kayseri, Turkey
- Nalan Özdemir, Department of Chemistry, Faculty of Sciences, Erciyes University, Talas Street, Kayseri, Turkey
- Narçın Palavan-Ünsal, Department of Molecular Biology and Genetics, Istanbul Kultur University, Atakoy Campus, Istanbul, Turkey
- Nazım Bozan, Department of Otorhinolaryngology, Faculty of Medicine, Yuzuncu Yil University, Van, Turkey
- Necip Ilhan, Department of Medical Biochemistry, Faculty of Medicine, Firat University, Elazig, Turkey
- Neel M. Fofaria, Department of Biomedical Sciences and Department of Immunotherapeutics and Biotechnology, Texas Tech University Health Sciences Center, Lubbock, USA
- Neriman İnanç, Department of Nutrition and Dietetics, Faculty of Health Sciences, University of Nuh Naci Yazgan, Kayseri, Turkey
- Nevin Çelebi, Department of Pharmaceutical Technology, Faculty of Pharmacy, Gazi University, Ankara, Turkey
- Nevin Ilhan, Department of Medical Biochemistry, Faculty of Medicine, Firat University, Elazıg, Turkey
- Nilgun Gurbuz, Department of Medical Biology, School of Medicine, Suleyman Demirel University, Isparta, Turkey
- Nilufer Cinkilic, Department of Biology, Science and Art Faculty, Uludag University, Bursa, Turkey
- Nur Selvi, Faculty of Medicine Department of Medical Biology, Ege University, Turkey
- Nurcan Silahtarlıoğlu, Graduate School Natural Applied Science, Erciyes University, Kayseri, Turkey
- Nurhayat Sutlupinar, Department of Pharmacognosy, Faculty of Pharmacy, Istanbul University, Istanbul, Turkey
- O. Faruk Kirlangic, Department of Molecular Biology and Genetic, Balikesir University, Balikesir, Turkey
- Oguzhan Tatar, Department of Medical Biochemistry, Faculty of Medicine, Firat University, Elazig, Turkey
- Oktay Özkan, Department of Medicinal Pharmacology, Faculty of Medicine, Niğde Ömer Halisdemir University, Niğde, Turkey
- Onur Bender, Biotechnology Institute, Ankara University, Ankara, Turkey
- Onur Kaya, Graduate School Natural Applied Science, Erciyes University, Kayseri, Turkey
- Oruc Allahverdiyev, Department of Pharmacology, Faculty of Pharmacy, Yuzuncu Yıl University, Van, Turkey
- Osman Oğuz, Molecular Biology and Genetics, Faculty of Life and Natural Sciences, Abdullah Gul University, Kayseri, Turkey
- Osman Tugay, Department of Biology Program of Botany, Faculty of Sciences, Selcuk University, Konya, Turkey
- Osman Üstün, Department of Pharmacognosy, Faculty of Pharmacy, Gazi University, Ankara, Turkey
- Oya Bozdağ-Dündar, Department of Pharmaceutical Chemistry, Faculty of Pharmacy, Ankara University, Ankara, Turkey
- Ozer Yılmaz, Department of Biology, Science and Art Faculty, Uludag University, Bursa, Turkey
- Ozgun Teksoy, Biology Department, Art and Science Faculty, Eskişehir Osmangazi University, Eskişehir, Turkey
- Ozgur Tag, Cancer Biology Laboratory, Department of Chemistry, Graduate School of Natural and Applied Sciences, Ege University, Izmir, Turkey
- Ozgur Vatan, Department of Biology, Science and Art Faculty, Uludag University, Bursa, Turkey
- Ömer Taş, Department of Pharmacognosy, Faculty of Pharmacy, Erciyes University, Kayseri, Turkey
- Özge Al, School of Medicine, Department of Anatomy, Erciyes University, Kayseri, Turkey
- Özge Güzel, Department of Bioengineering, Faculty of Engineering, Izmir Institute of Technology, Izmir, Turkey
- Özge Rencüzoğulları, Atakoy Campus, Department of Molecular Biology and Genetics, Istanbul Kultur University, Istanbul, Turkey
- Özlem Temiz-Arpacı, Department of Pharmaceutical Chemistry, Faculty of Pharmacy, Ankara University, Ankara, Turkey
- Pelin Taştan, Deparment of Pharmacognosy, Faculty of Pharmacy, Ege University, İzmir, Turkey
- Pelin Toros, Department of Histology & Embryology, Faculty of Medicine, Manisa Celal Bayar University, Manisa, Turkey
- Perihan Gürbüz, Pharmacognosy Dept., Faculty of Pharmacy, Erciyes University, Kayseri, Turkey
- Petek Ballar, Faculty of Pharmacy, Department of Biochemistry, Ege University, Izmir, Turkey
- Pınar Atalay Dündar, Department of Basic Sciences, Faculty of Pharmacy, Erciyes University, Kayseri, Turkey
- Pınar İkiz, Pharmacognosy Dept., Faculty of Pharmacy, Hacettepe University, Ankara, Turkey
- Pınar K. Sönmez, Department of Histology & Embryology, Faculty of Medicine, Manisa Celal Bayar University, Manisa, Turkey
- Pınar Kılıçaslan Sönmez, Department of Histology and Embryology, Faculty of Medicine, Celal Bayar University, Manisa, Turkey
- Pınar Obakan-Yerlikaya, Department of Molecular Biology and Genetics, Istanbul Kultur University, Atakoy Campus, Istanbul, Turkey
- Pınar Özden, Department of Medical Biology, Meram Faculty of Medicine, Necmettin Erbakan University, Konya, Turkey
- Pinar K. Sönmez, Department of Histology & Embryology, Faculty of Medicine, Manisa Celal Bayar University, Manisa, Turkey
- Rana Kavurmacı, Department of Advanced Technology, Ahi Evran University, Kırşehir, Turkey
- Randolph R. J. Arroo, Leicester School of Pharmacy, De Montfort University, The Gateway, Leicester LE1 9BH, UK
- Recep Eröz, Department of Genetics, Faculty of Medicine, Duzce University, Düzce, Turkey
- Remzi Soner Cengiz, Faculty of Veterinary Medicine, Turkey
- Remziye Kendirci, Department of Histology and Embryology, School of Medicine, Manisa Celal Bayar University, Manisa, Turkey
- Renata Francik, Department of Bioorganic Chemistry, Medical College, Jagiellonian University, Krakow, Poland
- Roderich D. Süssmuth, Institut für Chemie, Technische Universitat Berlin, Strasse des 17. Juni 124, Berlin, Germany
- Rojen Geylan, Department of Pharmacognosy, Faculty of Pharmacy, Erciyes University, Turkey
- Ruziye Daşkın, Botany Dept., Faculty of Science and Letters, Uludağ University, Bursa, Turkey
- S. Kerem Aydin, Sirri Yircali Anatolian High School, Balikesir, Turkey
- Sanjay K. Srivastava, Department of Biomedical Sciences and Department of Immunotherapeutics and Biotechnology, Texas Tech University Health Sciences Center, Lubbock, USA
- Seda Duman, Department of Bioengineering, Faculty of Engineering, Izmir Institute of Technology, Izmir, Turkey
- Seda Şirin, Gazi University, Faculty of Science, Department of Biology, Teknikokullar, Ankara, Turkey
- Sedat Ünal, Erciyes University Faculty of Pharmacy Department of Pharmaceutical Technology
- Seher Dalgic, Sirri Yircali Anatolian High School, Balikesir, Turkey
- Seher Yılmaz, School of Medicine, Department of Anatomy, Bozok University, Yozgat, Turkey
- Selda Eren, Faculty of Pharmacy, Erciyes University, Kayseri, Turkey
- Selen İlgün, Faculty of Pharmacy, Department of Pharmaceutical Botany, Erciyes University, Kayseri, Turkey
- Selim Demir, Department of Nutrition and Dietetics, Faculty of Health Sciences, Karadeniz Technical University, Trabzon, Turkey
- Sema Misir, Department of Biochemistry, Faculty of Pharmacy, Cumhuriyet University, Sivas, Turkey
- Senem Akkoç, Department of Chemistry, Faculty of Sciences, Erciyes University, Kayseri, Turkey
- Serap Yalcin, Department of Molecular Biology and Genetics, Ahi Evran University, Kırşehir, Turkey
- Serap Yalın, Department of Biochemistry, Faculty of Pharmacy, Mersin University, Mersin, Turkey
- Sevil Albayrak, Biology Department, Science Faculty, Erciyes University, Kayseri, Turkey
- Sevinç İnan, Department of Histology and Embryology, Faculty of Medicine, Celal Bayar University, Manisa, Turkey
- Sevtap Çağlar Yavuz, Department of Chemistry, Faculty of Sciences, Erciyes University, Kayseri, Turkey
- Seyhan Altun, Department of Biology, Faculty of Science, University of Istanbul, Istanbul, Turkey
- Sezin Anil, Department of Pharmacognosy, Faculty of Pharmacy, Istanbul University, Istanbul, Turkey
- Sharavan Ramachandran, Department of Biomedical Sciences and Department of Immunotherapeutics and Biotechnology, Texas Tech University Health Sciences Center, Lubbock, USA
- Sibel Gunes, Biology Department, Art and Science Faculty, Eskişehir Osmangazi University, Eskişehir, Turkey
- Sibel İlbasmiş Tamer, Department of Pharmaceutical Technology, Faculty of Pharmacy, Gazi University, Ankara, Turkey
- Sinem Yılmaz, Faculty of Pharmacy, Department of Biochemistry, Ege University, Izmir, Turkey
- Solmaz Susam, Department of Medical Biochemistry, Faculty of Medicine, Firat University, Elazig, Turkey
- Stefania Moccia, Institute of Food Sciences, National Research Council, Avellino, Italy
- Suheyl Furkan Konca, Department of Pharmaceutical Biotechnology, Faculty of Pharmacy, Erciyes University, Kayseri, Turkey
- Sukran Kultur, Department of Pharmaceutical Botany, Faculty of Pharmacy, Istanbul University, Istanbul, Turkey
- Sumeyra Cetinkaya, Medical Biology Department, Meram Medical Faculty, Necmettin Erbakan University, Konya, Turkey
- Suna Sabuncuoğlu, Toxicology Department, Faculty of Pharmacy, Hacettepe University, Ankara, Turkey
- Suna Sayğılı, Department of Histology and Embryology, Faculty of Medicine, Celal Bayar University, Manisa, Turkey
- Şamil Öztürk, Department of Histology & Embryology, Faculty of Medicine, Manisa Celal Bayar University, Manisa, Turkey
- Şebnem Kurhan, Novel Food Technologies Development, Application and Research Center, Abant İzzet Baysal University, Bolu, Turkey
- Taner Dağcı, Department of Physiology, Faculty of Medicine, Ege University, İzmir, Turkey
- Terken Baydar, Toxicology Department, Faculty of Pharmacy, Hacettepe University, Ankara, Turkey
- Tevhide Sel, Ankara Unıversıty, Faculty of Veterinary Medicine, Turkey
- Tolga Cavas, Medical Biology Department, Faculty of Medicine, Uludag University, Bursa, Turkey
- Tolga Ertekin, School of Medicine, Department of Anatomy, Kocatepe University, Afyon, Turkey
- Tuna Onal, Department of Histology & Embryology, Faculty of Medicine, Manisa Celal Bayar University, Manisa, Turkey
- U. Sebnem Harput, Department of Pharmacognosy, Faculty of Pharmacy, Hacettepe University, Ankara, Turkey
- Ünal Egeli, Medical Biology Department, Faculty of Medicine, Uludag University, Bursa, Turkey
- Varol Sahinturk, Vocational School of Health Services, Eskişehir Osmangazi University, Eskişehir, Turkey
- Vasilis P. Androutsopoulos, Department of Toxicology, Medical School, University of Crete, Crete GR, Greece
- Veysel Kayser, Faculty of Pharmacy, The University of Sydney, Sydney, Australia
- Vildan Betül Yenigun, Department of Medical Biochemistry, Faculty of Medicine, Bezmialem Vakif University, Turkey
- Yalcin Erzurumlu, Faculty of Pharmacy, Department of Biochemistry, Ege University, Izmir, Turkey
- Yasemin Tekin, Biology Department, Art and Science Faculty, Eskişehir Osmangazi University, Eskişehir, Turkey
- Yasin Genc, Department of Pharmacognosy, Faculty of Pharmacy, Hacettepe University, Ankara, Turkey
- Yeşim Aktaş, Erciyes University Faculty of Pharmacy Department of Pharmaceutical Technology
- Yuksel Aliyazicioglu, Medicinal Plants, Traditional Medicine Practice and Research Center, Gumushane University, Gumushane, Turkey
- Yüksel Öğünç, Department of Biochemistry, Faculty of Pharmacy, Anadolu University, Eskişehir, Turkey
- Zerrin Seller, Department of Biochemistry, Faculty of Pharmacy, Anadolu University, Eskişehir, Turkey
- Zeynep Dogan, Department of Pharmacognosy, Faculty of Pharmacy, Hacettepe University, Ankara, Turkey
